# *In vivo* imaging of adipose-derived mesenchymal stem cells in female nude mice after simulated childbirth injury

**DOI:** 10.3892/etm.2014.2092

**Published:** 2014-11-27

**Authors:** MIAO DAI, PEIRONG XU, MIN HOU, YINCHENG TENG, QINGKAI WU

**Affiliations:** Department of Obstetrics and Gynecology, Shanghai Jiao Tong University Affiliated Sixth People’s Hospital, Shanghai 200233, P.R. China

**Keywords:** adipose-derived mesenchymal stem cells, bioluminescence imaging, simulated childbirth injury

## Abstract

The aim of the present study was to track *in vivo* the distribution and survival of adipose-derived mesenchymal stem cells (ASCs) transplanted into female BALB/c nude mice following simulated childbirth injury, using green fluorescent protein and luciferase dual labeling, bioluminescent imaging (BLI) and histological evaluation. The results demonstrated that the dually labeled ASCs could be detected for up to eight weeks *in vivo*. The number of implanted cells decreased during the first three weeks, and then stabilized until the end of the experiment. According to the linear regression plot, ~27,621 implanted cells survived until eight weeks after implantation. Transplanted ASCs predominantly existed at the inoculation site of the vagina, with little or no spread to other organs. Histological analysis confirmed the survival of the engrafted ASCs. The study provided basic evidence that BLI techniques can be used to monitor ASCs *in vivo* in real time and in the long term. Through local administration, ASCs could survive in the long term to facilitate repair following pelvic-floor injury.

## Introduction

Vaginal childbirth can cause pelvic injury, which can lead to pelvic-floor dysfunction (PFD) (1). During vaginal delivery, the fetal head pressure on the pelvic floor results in direct trauma to the pelvic organs (2). These injuries can lead to the development of pelvic-floor disorders, including stress urinary incontinence (SUI) and fecal incontinence (3). The available therapeutic options for PFD include a variety of methods, such as pelvic-floor muscle exercise, electrical stimulation, pessaries and surgical therapy. Although several treatments exist, no current therapy can fully repair the underlying pathophysiology (4–5).

Recently, cell-based therapy has gained attention as a potential treatment for PFD, particularly for SUI. Numerous animal and clinical studies (6–8) have demonstrated the potential of such therapies to bring about functional and anatomical improvements. Adipose-derived mesenchymal stem cells (ASCs) have the same capacity for self-renewal and differentiation as bone marrow mesenchymal stem cells (BMSCs). Furthermore, they can be easily harvested by a simple, minimally invasive method and they are more abundant than BMSCs (9). ASCs possess the ability to facilitate the synthesis of new collagen and the potential to differentiate into smooth muscle cells, which constitute the pelvic floor (10). Based on this, ASCs are considered as ideal adult stem cells to treat PFD (11); however, the length of their survival and their distribution in living animals remains unknown.

The aim of this study was to apply advanced imaging systems to aid in the development of cell therapy for PFD. The fate and distribution of ASCs in nude mice were tracked *in vivo* in real time following vaginal balloon dilation, synergizing with non-invasive bioluminescent optical imaging.

## Materials and methods

### Isolation and culture of rabbit ASCs

All animal procedures were performed under guidelines approved by the Institutional Animal Care and Use Committee (Shanghai Jiao Tong University Affiliated Sixth People’s Hospital, Shanghai, China). ASCs were obtained from adipose tissue in the inguinal fat pads of a New Zealand white male rabbit (The Animal Institute, School of Medicine, Shanghai Jiao Tong University, Shanghai, China). The fat pads were washed three times in phosphate-buffered saline containing 1% penicillin/streptomycin (P/S; Gibco Life Technologies, Beijing, China) and then digested with 5 ml collagenase I (1 mg/ml; Gibco). After 60 min of digestion in a shaking incubator (BS-4G, Changzhou Feipu Experimental Instrument Factory, Beijing, China) at 200 rpm and 37°C, 8 ml high-glucose Dulbecco’s Modified Eagle Medium (DMEM; Gibco), 10% fetal bovine serum (FBS; Sigma, St Louis, MO, USA) and 1% P/S were added to terminate digestion. The cells were sieved through a 70-μm cell strainer (Shanghai Jun Sheng Biological Technology Co., Ltd., Shanghai, China) followed by centrifugation at 400 × g for 15 min. The cell-containing pellets were seeded into a 100-mm^2^ cell culture dish in the presence of complete media (DMEM + 10% FBS + 1% P/S) at 37°C in a 5% CO_2_ incubator (Thermo Electron Corporation, Waltham, MA, USA). Media were changed every three days to remove nonadherent cells and tissue debris.

### Lentiviral infection

The lentiviral vectors (SunBio Biotech, Beijing, China) contained the enhanced green fluorescent protein (eGFP) and Luciferase (Luc) genes. For infection, the ASCs at passage 3 were seeded in a six-well cell culture cluster, infected with concentrated lentivirus particle stock (1×10^7^ transduction U/ml; multiplicity of infection, 50) when 70–80% confluence was achieved, and then incubated in medium with 10 μg/ml polybrene (Sigma) for 24 h. The ASCs infected with recombinant lentivirus were selected and purified by puromycin (1 μg/ml; SunBio Biotech) and the transduction efficiency of the ASCs was evaluated by flow cytometry (FACS Calibur™ flow cytometer, BD Biosciences, Franklin Lanes, NJ, USA).

### Cell proliferation assay

Cell viability subsequent to infection was measured by cell-counting kit (CCK)-8 assays. At the desired time-points, the (eGFP + Luc)-ASCs at passage 6 were incubated in CCK-8 solution (Dojindo, Kumamoto, Japan) at 37°C in a 5% CO_2_ incubator for 3 h. The absorbance of the supernatants was measured at a wavelength of 450 nm.

### Luc assays

To determine Luc activity *in vitro*, different quantities of (eGFP + Luc)-ASCs (10, 100, 500, 1,000, 5,000 and 10,000)/100 μl at passage 6 were seeded onto black, clear-bottom 96-well plates in 100 μl medium (DMEM + 10% FBS + 1% P/S), respectively. A row of wells was seeded with unlabeled cells as controls. To every 100 μl culture medium (in a 96-well plate) was added 15 μl D-luciferin (Sciencelight, Shanghai, China) solution (150 μl/ml). A highly sensitive, cooled charge-coupled device (CCD) camera (Xenogen IVIS; Xenogen, Hopkinton, MA, USA) was used to detect the bioluminescent signals. Quantification of the signals was performed by the acquisition and analysis software Living Image (Xenogen).

To establish the correlation between the number of transplanted cells and the light produced *in vivo*, serial dilution of (eGFP + Luc)-ASCs (1×10^6^/100 μl, 1×10^5^/100 μl, 1×10^4^/100 μl, 1×10^3^/100 μl and 1×10^2^/100 μl) were grafted in the subcutaneous space in the dorsum of each nude mouse. The animals were intraperitoneally injected with D-luciferin (150 mg/kg) and anesthetized with isoflurane, prior to being directly imaged by CCD. Quantification of the signals was performed by the software Living Image (Xenogen).

### Vaginal distention (VD) and cell inoculation

Twenty female BALB/c nude mice (The Animal Institute, School of Medicine) at five weeks old (16±1.0 g) were used in this study in accordance with the National Research Council Guide for the Care and Use of Laboratory Animals. Mice were anesthetized by intraperitoneal injection of sodium pentobarbital (30 mg/kg; Shanghai Yuanye Bio-Technology Co., Ltd, Shanghai, China), prior to undergoing a simulated childbirth injury by VD. A modified 8-Fr Foley catheter was inserted into the vagina and the balloon was inflated to 2.5 ml for 4 h. One hour after injury, 1×10^6^ labeled ASCs at passage 9 resuspended in 50 μl medium were injected into the vaginal submucosa of 10 mice. To act as a control, a further 10 mice were injected in the same way with cell-free media.

### In vivo non-invasive bioluminescence imaging (BLI) and image quantification

At 1 h and one, two, three, four, six and eight weeks after VD, mice were anesthetized (1–3% isoflurane; Science and Technology Development Co., Ltd., Shenzhen City Rui Bolong, Guangdong, China), and given the substrate D-luciferin by intraperitoneal injection at 150 mg/kg. The mice were then placed in a light-tight camera box with continuous exposure to 1–3% isoflurane. BLI signals were detected by the IVIS camera system, integrated, digitized and displayed. Quantification of the signals *in vivo* was performed by the software Living Image (Xenogen).

### Histology

To confirm the survival of engrafted ASCs, the entire vagina, heart, lung and liver were harvested as frozen sections for histological analysis at 8 weeks after VD. Sections (4-μm thick) were stained by DAPI, followed by detection of eGFP-positive cells by fluorescence microscopy (Leica AF6000, Leica, Wetzlar, Germany).

### Statistical analyses

Quantitative values are expressed as the mean ± standard error of the mean. Regression plots were used to describe the association between bioluminescence and cell number. R^2^ values were reported to assess the quality of the regression model. P<0.05 was considered to indicate a statistically significant difference.

## Results

### Isolation, culture and eGFP expression efficiency of ASCs

**Figure 1:**
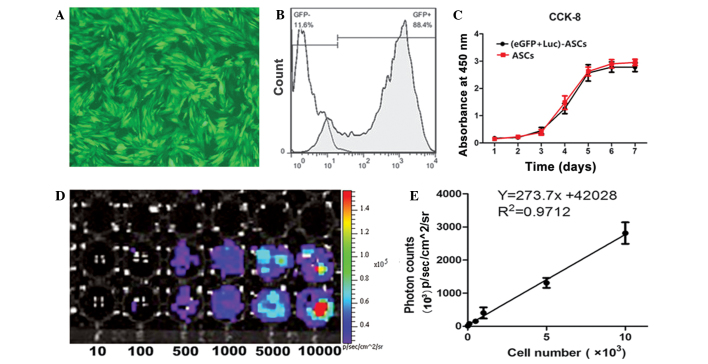
(A) Morphologies and eGFP expression of ASCs at passage 6 (fluorescence microscopy; magnification, ×10). (B) Transduction efficiency of (eGFP + Luc)-labeled cells at passage 6. The count of unlabeled cells is shown through the white area and that of eGFP-positive cells through the gray area. (C) (eGFP + Luc)-labeled cell proliferation in the CCK-8 assay. (D) *In vitro* bioluminescence imaging of serial dilution of (eGFP + Luc)-ASCs (10 to 10,000)/100 μl. The color bar indicates the light production level: Blue, lowest intensity; Red, highest intensity. (E) Plots of light production extracted from the images versus the number of (eGFP + Luc)-ASCs. The slope of the linear regression plot was 273.7±14.91 for the ASCs. R, correlation coefficient; eGFP, enhanced green fluorescent protein; Luc, luciferase; CCK8, cell-counting kit-8; ASC, adipose-derived mesenchymal stem cells.

ASCs showed high proliferation rates and adherence to plastic surfaces. The spindle-shaped adherent cells grew and reached confluence in 5–7 days. ASCs labeled with eGFP and Luc genes had a high eGFP expression level and were selected and purified by puromycin for use in the subsequent experiments (Fig. 1A). The transduction efficiency of the ASCs at passage 6 was up to 88.4%, as shown by flow cytometry (Fig. 1B). The CCK-8 assay was used to evaluate the viability of the ASCs following transduction. No significant differences were observed between the unlabeled and eGFP + Luc-labeled ASCs (Fig. 1C).

### Light production capacity of (eGFP + Luc)-ASCs at passage 6 in vitro

The light production capacity of (eGFP + Luc)-ASCs at passage 6 *in vitro* was measured in lysates from predetermined numbers (10, 100, 500, 1,000, 5,000 and 10,000) of (eGFP + Luc)-ASCs. A minimum of 100 (eGFP + Luc)-ASCs were required for cell detection with the imaging system (Fig. 1D). Luc was expressed *in vitro* steadily. The slopes of the linear regression plots showed standard plots of light production versus cell number (R^2^=0.9712). The slope of the linear regression plot was 273.7±14.91 for the ASCs (Fig. 1E) (slope of regression plot = number of relative light units/cell). The number of (eGFP + Luc)-ASCs was linearly correlated with light production, indicating that the BLI signals could be used to quantitatively track labeled ASCs.

### In vivo non-invasive imaging for implanted (eGFP + Luc)-ASCs

**Figure 2:**
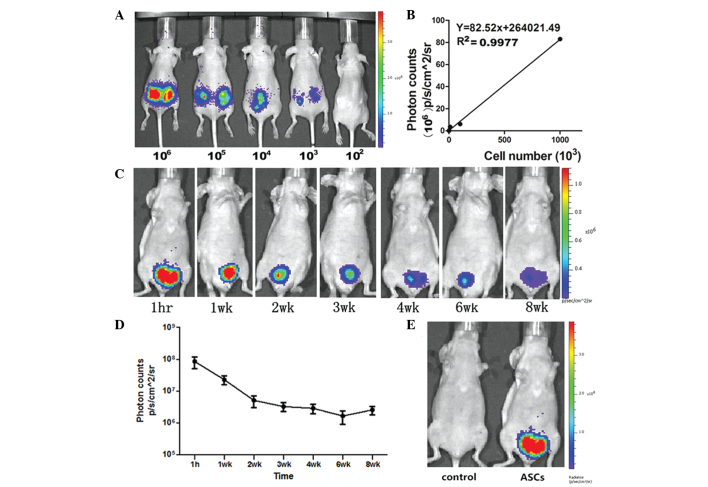
BLI analysis. (A) *In vivo*, mice were transplanted with predetermined numbers (1×10^2^ to 1×10^6^) of (eGFP + Luc)-ASCs in each dorsum and used for imaging. (B) Light production versus the number of transplanted cells in vivo was linear (R^2^=0.9977). The slope of the linear regression plots was 82.52. (C) BLI analysis of inoculated ASCs in pelvic-floor injury mice. Luc-expressing cells were inoculated in the pelvic-floor injury mice, and images were recorded at the indicated times. (D) BLI signals were extracted from the analysis software Living Image, and plotted versus the elapsed time post-inoculation. (E) BLI signals of the ASCs were detected 1 h after inoculation. No visible signal was observed in the control group. The color bar shows the levels of light produced by the inoculated ASCs, and the arbitrary color bar and numbers illustrate relative light intensities from the lowest (blue) to the highest (red). BLI, bioluminescence imaging; ASC, adipose- derived mesenchymal stem cell; eGFP, enhanced green fluorescent protein; Luc, luciferase.

To quantify the implanted (eGFP + Luc*)*-ASCs *in vivo*, five mice were implanted with predetermined numbers of (eGFP + Luc)-ASCs (1×10^2^, 1×10^3^, 1×10^4^, 1×10^5^ and 1×10^6^) at the same time to establish the correlation between the number of transplanted cells and the light produced (Fig. 2A). The slope of the linear regression plots was 82.52 (R^2^=0.9977) (Fig. 2B).

Following cell inoculation in the VD mice, the mice were imaged longitudinally (1 h, one, two, three, four, six and eight weeks after inoculation) to analyze their light-producing behavior. The average BLI signals in mice were as follows: 1 h post-inoculation, (8.49±6.01)x10^7^ p/sec/cm^2^/sr; 1 week post-inoculation, (2.26±1.20)x10^7^ p/sec/cm^2^/sr; 2 weeks post-inoculation, (5.07±3.51)x10^6^ p/sec/cm^2^/sr; 3 weeks post-inoculation, (3.24±1.74)x10^6^ p/sec/cm^2^/sr; 4 weeks post-inoculation, (2.85±1.65)x10^6^ p/sec/cm^2^/sr; 6 weeks post-inoculation, (1.64±1.28)x10^6^ p/sec/cm^2^/sr; and 8 weeks post-inoculation, (2.54±1.33)x10^6^ p/sec/cm^2^/sr. The BLI signals of the ASCs predominantly existed at the inoculation site of the pelvic organ, and no visible signals were observed in other organs such as the lung, heart and liver. During the first three weeks, BLI signals markedly decreased. The signals were then observed to stabilize during the remaining weeks of the experiment. A total of 97% of transplanted cells were lost at 12 weeks after transplantation, and ~27,621 of the implanted cells survived according to the linear regression plot. Visible signals were still observed in mice injected with (eGFP + Luc)-ASCs by the end of the experiment (Fig. 2C and D). No visible signal was observed in the control group (Fig. 2E).

### Histology

**Figure 3:**
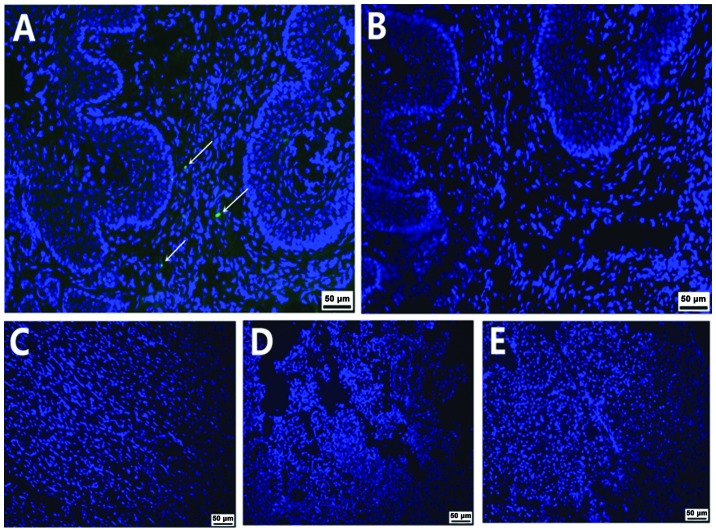
DAPI staining for eGFP-positive cells eight weeks after transplantation. (A and B) Fluorescence microscopy (magnification, ×20) of vaginal tissue from the (A) ASC transplantation group and (B) control group. GFP was expressed around the nuclei dyed by blue DAPI (arrowheads). (C, D and E) Fluorescence microscopy (magnification, ×10) of tissue from the (C) heart, (D) lung and (E) liver of the transplantation group. Internal scale marker=50 μm; eGFP, enhanced green fluorescent protein; ASC, adipose-derived mesenchymal stem cell.

To identify if the implanted cells had survived, the tissues of the vagina and other organs in the transplantation group were harvested for DAPI staining at eight weeks after transplantation. eGFP-positive ASCs were detected at the submucous layer of the vagina (Fig. 3A). No eGFP-positive cells were detected in the control group (Fig. 3B) or other organs, such as the heart, lung and liver (Fig. 3C, D and E).

## Discussion

Cell-based therapy has shown the potential to improve function and anatomy in PFD; however, no study has been conducted regarding the survival and distribution of ASCs in pelvic-floor injury *in vivo* in real time. In the present study, a non-invasive BLI-based approach was used to analyze *in vivo* the behavior of eGFP + Luc-expressing ASCs in model nude mice with pelvic-floor injury.

The ability to detect ASCs early on in live animals and in real time is ideal for accurately monitoring the behavior of ASCs. There are different methods to track the stem cells *in vivo*. Previous studies tracked the transplanted ASCs in pelvic-floor injury models using bromodeoxyuridine and CellTracker™CM-Dil (12–14). Although this method is relatively straightforward, a significant weakness is that the fluorescence intensity decreases during cellular proliferation *in vivo*. Furthermore, the technique cannot monitor the inoculated cells in real time. The present study monitored the labeled cells implanted in mice for >8 weeks *in vivo* in real time by a non-invasive BLI-based approach. This period was longer than that in a previous study (8), which may be attributable to i) the high and stable transduction efficiency and ii) BLI. Firstly, lentiviral vectors could label both dividing and non-dividing cells, and the transduction efficiency of the ASCs was up to 88.4%. Vectors are a high-performance tool for labeling ASCs in order to detect the majority of the transplanted cells. Secondly, based on the generation of visible light photons by Luc reporters introduced into live cells, BLI techniques provide excellent signal-to-noise ratios due to the low intrinsic bioluminescence of mammalian tissues. BLI can be used to assess the viability of stem cells *in vivo* in real time, facilitating the development of stem cell therapy.

The transplanted cells encountered mass death within three weeks after transplantation. This phenomenon was consistent with a previous report (15) and may have resulted from i) limited survival space in the pelvic-floor tissues following injection; ii) cell necrosis; and iii) inflammation and immune reactions following transplantation, leading to cell loss. The repair functions of stem cells rely on the population of stem cells (16). Consequently, it is of great importance to augment the number of implanted ASCs and diminish cell loss. Although 97% of transplanted cells were lost, ~27,621 implanted cells survived in the pelvic floor. A previous study also indicated that the transplantation of ASCs could improve urethral function (13). It remains unknown how the transplanted ASCs function, although we propose that the local injection of ASCs could promote recovery by an atrophic factor mechanism that modifies cellular and extracellular elements of pelvic organs.

The BLI signals of ASCs predominantly existed at the inoculation site of the pelvic organ, and no visible signals were observed in other organs, such as the lung, heart and liver, which suggested that the optimal location of ASCs via local administration is at the injury site. Implanted cells had little or no tendency to migrate to other organs. The reasons may be that i) local administration augments the number of transplanted cells at the injury site; ii) the injury site releases chemokines that can attract ASCs via a cytokine gradient (17–19); iii) the long-term survival of stem cells could facilitate differentiation into vessel cells to maintain cell nutrition and reduce the cell loss at the inoculation site. Although intravenous injection is less invasive, a limited number of implanted cells home to the injury site, and the majority of the cells are held in the lungs (15). It is therefore more effective to use local administration than intravenous administration. Future studies are likely to be focused on the optimal delivery of the cells.

One potential limitation is that the molecular mechanisms of the recovery of ASCs have not yet been clearly elucidated. Further research is required to determine the mechanism of accelerated recovery of cell-based therapies. Experiments to solve the growth crisis should be a focus in our future studies.

In conclusion, bioluminescence imaging could be used to monitor ASCs *in vivo* in real time, and the local administration of ASCs following simulated childbirth injury could allow the long-term survival of the cells at the inoculating site despite mass cell death, providing evidence of the potential for cell-based therapy to treat pelvic-floor injury.
